# Oleuropein Mitigates Acrylamide-Induced Nephrotoxicity by Affecting Placental Growth Factor Immunoactivity in the Rat Kidney

**DOI:** 10.5152/eurasianjmed.2023.23043

**Published:** 2023-10-01

**Authors:** Kıymet Kübra Tüfekci, Musa Tatar

**Affiliations:** 1 Department of Histology and Embryology, Kastamonu University Faculty of Medicine, Kastamonu Türkiye; 2 Department of Histology and Embryology, Kastamonu University Faculty of Veterinary Medicine, Kastamonu Türkiye

**Keywords:** Stereology, nephrotoxicity, placental growth factor, Cavalieri volume estimation

## Abstract

**Objective:**

Oleuropein is one of the main components of the antioxidant properties of olive leaves. Placental growth factor is an important regulator in angiogenesis and inflammation, its levels being variable in pathological conditions. In this study, we aimed to examine changes in placental growth factor expression and the effect of oleuropein, found in olive leaves, in rats exposed to acrylamide nephrotoxicity.

**Material and Methods:**

Twenty-four male Wistar albino rats were allocated into 4 groups. The control group received saline solution only. The oleuropein group received oleuropein (4.2 mg/kg), the acrylamide group received acrylamide (5 mg/kg), and the acrylamide and oleuropein group received acrylamide (5 mg/kg) and oleuropein (4.2 mg/kg). All substances were administered via gastric gavage for 21 days. Kidney tissues were removed at the end of the study and subjected to histopathological, stereological, and immunohistochemical procedures.

**Results:**

Histopathological examination revealed dilatation, vacuolization, and degeneration in the proximal and distal tubules and increased placental growth factor immunoreactivity in the acrylamide group. Cavalieri volume analysis revealed increased cortex, distal, and proximal tubule volumes (*P* < .01).

**Conclusion:**

Oleuropein significantly attenuated acrylamide-induced kidney injury by altering placental growth factor immunoreactivity. Placental growth factor immunoreactivity can be used as a marker of acrylamide nephrotoxicity, and oleuropein may counteract acrylamide-induced kidney injury.

**Main Points**

- Acrylamide, produced by heating foods with high carbohydrate content, causes severe renal histopathological changes.
- An increase in the placental growth factor may be a biomarker of kidney toxicity.
- Oleuropein exhibited a partial protective effect against acrylamide nephrotoxicity.

## Introduction

Widespread consumption of heat-treated foods results in a rapid increase in exposure to acrylamide (ACR), a group 2A carcinogen. A previous rat study showed that ACR is wholly and rapidly absorbed by the gastrointestinal system and distributed to peripheral tissues shortly after consuming foods with a high ACR content.^1^ Human studies have proved that this harmful substance passes from the placenta and milk to the infant.^2,3^ Additionally cooking methods, the ingredients contained, cooking temperature and time, and storage conditions (pH and humidity) also affect the formation of ACR. Moreover, ACR exhibits genotoxic, carcinogenic, neurotoxic, and nephrotoxic effects.^4^

A mouse study determined that different doses (10 mg, 20 mg, and 30 mg) of ACR resulted in DNA damage and chromosome abnormalities in bone marrow cells.^5^ The mechanism underlying the neurotoxic effects of ACR has been associated with kinesin-related motor proteins. This mechanism may also have similar implications for other deleterious effects detected in animals. In addition, due to the high affinity of ACR to sulfhydryl groups in proteins, it can inactivate proteins/enzymes that play a role in DNA repair and thus exhibit carcinogenic and neurotoxic effects.^6^ Furthermore, animal studies of the effects of ACR on kidney tissue have determined that degeneration in tubule and glomerular cells is caused by ACR-induced oxidative stress.^7,8^ More specifically, ACR exposure has been reported to promote apoptosis and significantly increase renal caspase-3 expression.^9^

The placental growth factor (PlGF), a member of the vascular endothelial growth factor (VEGF) family, is essential in pathological conditions.^10^ Since kidney tissue is highly vascularized, maintaining the structure of its two highly important microvessels (glomerular and peritubular) is critical in terms of function. The VEGF family, which acts a vital role in the maintenance of kidney functions, is therefore the primary regulator of blood vessel growth, promoting the survival of the endothelium and protecting the microvasculature.^11^ It has been found that patients with chronic kidney disease have a significant elevate in plasma PlGF levels.^12^ An animal model study also reported a higher messenger ribonucleic acid (mRNA) level of PlGF in kidney tissue.^13^ Although the pathophysiological role of VEGF in various renal disease models has been frequently investigated,^14,15^ there have been no studies to date concerning PlGF expression in exposure to ACR.

It has been identified as a potential antioxidant molecule in olive oil leaves.^16^ Oleuropein (OLE) exhibits powerful antioxidant activities by donating hydrogen to prevent oxidation, thanks to the hydroxyl groups (especially the 1,2-dihydroxybenzene part) in its chemical structure.^17^ In an experimental model of acute kidney injury, OLE was reported to suppress oxidative, inflammatory, and apoptotic responses and to reverse molecular, biochemical, and histological changes in the kidney.^18^ Oleuropein has been reported to be a potential antiangiogenic agent in breast cancer cells.^19^

To our knowledge, no studies have addressed the role of PlGF in ACR-induced renal damage and the therapeutic effect of OLE. The present research therefore aimed to examine the expression of PlGF as a key molecule for angiogenesis against ACR nephrotoxicity and to evaluate the potential effects of OLE treatment.

## Materials and Methods

The experiments were conducted with 24 male Wistar albino rats (12 weeks old, 220 ± 50 g). These were kept at 24 ± 1°C in a 12 : 12-hour light/dark cycle during the study. Food and water were available ad libitum. The animal experiments in this study were conducted in accordance with the current regulations of the Ethics Committee in the Balıkesir University Experimental Animals Research Center. Approval study was also granted by the ethical committee (Ethical approval number: 2022/10-1). Animal welfare and ethical principles were observed in all procedures performed on the experimental animals. The amount of OLE (4.2 mg/kg) in olive leaf extract (100 mg/mL)^20^ was determined using the high-performance liquid chromatography method.^21^ Olive leaves were obtained from olive groves in Balikesir in the South Marmara region of Turkey. The ARRIVE guidelines 2.0 (Animal Research: Reporting of In Vivo Experiments) were strictly followed while doing this experiment.

### Study Groups

1: Control group (Cont, n = 6)
The rats received saline solution by oral gavage for 21 days.
2: Oleuropein group (OLE, n = 6)
The rats received OLE by oral gavage (4.2 mg/kg) for 21 days.
3: Acrylamide group (ACR, n = 6)
The rats in this group received ACR (BioShop, Cat No:79.06.1) by oral gavage (5 mg/kg dissolved in saline solution) for 21 days.
4: Acrylamide + oleuropein group (ACR + OLE, n = 6)

The rats in this group received both ACR (5 mg/kg) and OLE (4.2 mg/kg) by oral gavage for 21 days.

At the end of the 21 days of the experiment, all animals were anesthetized by ketamine (50 mg/kg) and xylazine (10 mg/kg) intraperitoneal injection and then sacrificed by cervical dislocation. The removed tissues were immediately immersed in 10% neutral-buffered formalin, after which they were subjected to routine tissue processing procedures (washing, dehydration, clearing, and embedding in paraffin blocks).

### Hematoxylin–Eosin Staining

Five-micrometer thick sections were taken according to the sampling rule (systematic randomly, 1/250) for histopathological and stereological analysis. Sections were stained in line with the hematoxylin-eosin staining procedure.

### Immunohistochemical Analysis

In order to examine the immunohistochemistry of kidney tissues, sections were first deparaffinized and then treated with 3% H_2_O_2_. The sections were then rinsed with phosphate-buffered saline solution for 15 minutes. After, the antigen retrieval procedure was applied and then incubated in a 10% goat serum-blocking solution (TP-125-HL, Ultra V Block®, Thermo Fisher Scientific, Fresno, Calif, USA). The primary antibody (anti-PlGF, Novus Biologicals, Centennial, Colo, USA, Cat no: NBP2-67067, dilution 1:250) was dropped into the sections for 1.30 hours. The slides were subsequently incubated with mouse and rabbit specific HRP complex (ABCAM, ab236466) and stained with a 3,3’-Diaminobenzidine (DAB) chromogenic solution. Next, they were counterstained with Gill’s hematoxylin and covered with mounting medium. Finally, PlGF-immunopositive cells in the kidney were evaluated using a microscope (Zeiss Axiolab 5, Jena, Germany).

Semiquantitative analyses of PlGF immunoreactivity were applied using histochemical scoring (H-score) assessment.^22^ The immunohistochemical staining intensity was considered as either weak, medium, strong, or very strong.^22^ Finally, the immunoreactivity score is obtained by multiplying the staining intensity by the percentage of immunoreactive cells.

### Stereological Analysis

**Figure 1.**
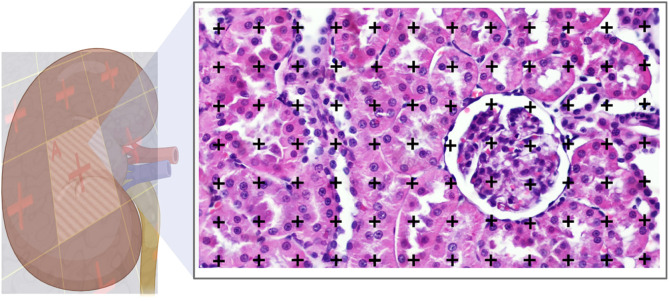
Application of the “point counting method” for kidney tissue volume estimation. In this method, the point probe is randomly placed on each section, and the points that intersect with the structures of interest (distal and proximal tubules, glomeruli, etc.) are counted.

Cavalieri volume calculation was used to determine quantitative changes in kidney tissue. According to the Cavalieri method, the total surface area of the structure of interest is found in the sections and multiplied by the section thickness. For this purpose, a point grid was put over the kidney-section images, and the surface area was calculated. Field images of tissue sections were captured on a 20× objective lens (Zeiss Axiolab 5). The tissue sampling interval was determined as (1/8). The area between the points placed on the section was determined as 25 600 µm^2^. The points that intersected with the cortex, medulla, proximal, distal tubules, and glomeruli were counted. Volume analysis was carried out on ImageJ software (Java image processing program, NIH, Bethesda, Maryland, USA)^23^ (Figure 1).

The volumes of the structures in each section were estimated using the following equation:



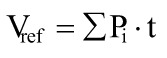





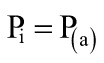



(*V_ref_:* Reference volume; ΣP*_i_*: Total number of points intersecting the section; *P_(a)_*: The area represented by a point; *t*: Section thickness).

### Statistical Analysis

Statistical Package for the Social Sciences software SPSS version 26.0 (IBM SPSS Corp.; Armonk, NY, USA) was used for statistical analysis of data. The data were expressed as mean ± standard error. Data were compared using 1-way analysis of variance followed by Tukey’s multiple comparison test. *P*-values less than .05 and .01, as applicable, were regarded as statistically significant.

## Results

### Histopathological Results

Histopathological examination of the kidneys revealed a normal histological appearance in the cortex and medulla regions in sections from the Control and OLE groups. 

In the ACR-exposed kidney tissue, however, severe vacuolization was detected in the proximal tubules. Tubular dilatation and some necrotic epithelial cells were observed in the ACR group distal tubules. Damaged areas were also noted, especially in the tubular, vascular, and interstitial regions in the sections from this group. Vascular occlusion with the expansion of interstitial capillaries was also observed. This may indicate impaired blood flow in the end capillary bed. Leukocyte infiltration was detected in some areas near the renal corpuscle.

**Figure 2.**
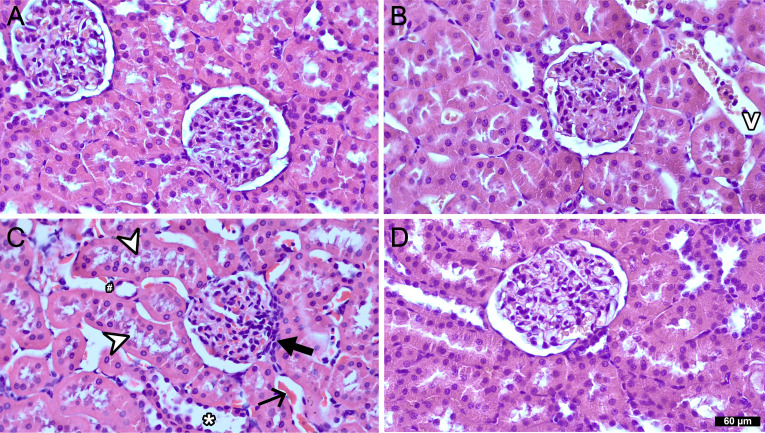
(A–D) Histopathological examination of H&E-stained kidney tissues from the Control, OLE, ACR, and ACR + OLE groups, respectively. (A, B) The kidneys from the control and OLE groups exhibited a normal histological structure in the tubules, renal corpuscle, and blood vessels (v). (C) Interstitial vascular occlusion (thin arrow) was detected in sections from the ACR group, together with tubule vacuolization (arrowhead), tubule enlargement (asterisk); leukocyte infiltration (thick arrow), and necrotic tubule cells (#). (D) However, the normal histological structure of the kidneys from the ACR + OLE group was preserved, and the tubules, renal corpuscle, and vascularization were also normal. Scale bar; 60 µm for all panels. ACR, acrylamide; OLE, oleuropein.

In the sections from the ACR + OLE group, the general histological structure was preserved, and the blood vessels and tubules exhibited a normal appearance (Figure 2).

### Immunohistochemical Results

**Figure 3.**
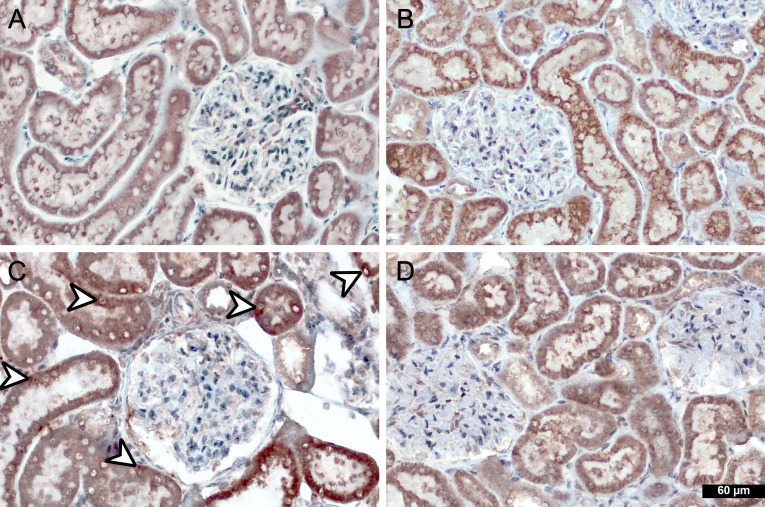
(A–D) Immunohistochemical anti-PlGF staining in kidney tissues from the Control, OLE, ACR, and ACR + OLE groups. Diffuse positive PlGF cytoplasmic staining was detected in sections from all 4 groups. (C) In the ACR group, the arrowheads point to an example of epithelial cells with positive nuclear staining, which was more frequently seen with ACR exposure. Scale bar; 60 µm for all panels. ACR, acrylamide; OLE, oleuropein.

Diffuse PlGF immunoreactivity was observed in kidney tissues from all the groups (Figure 3). The PlGF-immunopositive cells stained more intensely and H-scores were higher in the ACR group compared to the other groups (*P* < .01). However, anti-PlGF staining intensity and H-scores in the ACR + OLE group were lower than those in the ACR group (*P* < .01), but there were no significant differences between the ACR + OLE and OLE groups (*P* > .05) (Tables 1 and 2).

### Stereological Results

#### Distal Tubule Volume

Total distal tubule volume (mm^3^) increased in the OLE group (*P* < .05), and significant elevation was also found in the ACR group compared to the Control (*P* < .01). A significant reduction in distal tubule volume was observed in the ACR + OLE group compared to the ACR group. Additionally, there was no difference between the ACR + OLE group and the Control group in terms of distal tubule volumes (*P* > .05) (Table 3).

#### Proximal Tubule Volume

Proximal tubule volume (mm^3^) differed significantly between the ACR group and the Control group (*P *< .01). There was no difference in proximal tubule volumes was observed between the OLE and Control groups (*P* > .05). While proximal tubule volume decreased in the ACR + OLE group compared to the ACR group (*P* < .01), it was no different to that in the Control group (*P* > .05) (Table 3).

#### Glomeruli Volume

There were no significant differences in glomeruli volumes in any of the groups (*P* > .05) (Table 3).

#### Cortex Volume

The volume of the cortex (mm^3^) increased significantly in the OLE, ACR, and ACR + OLE groups (*P* < .01). Oleuropein group cortex volume was lower than that in the ACR + OLE group (*P* < .05). Cortex volume in the ACR group was significantly higher than that in the OLE group (*P* < .01), but no significant difference was determined with the ACR + OLE group (*P* > .05) (Table 3).

#### Medulla Volume

Medulla volume (mm^3^) was significantly lower in the OLE and ACR groups (*P* < .01). No difference was observed between the OLE group and the ACR or ACR + OLE groups (*P* > .05). Medulla volume in the ACR + OLE group was significantly higher than in the ACR group (*P* < .01) but was no different to that in the Control group (*P* > .05) (Table 3).

## Discussion

Exposure to ACR before and after birth has been reported to cause neurotoxic, genotoxic, and carcinogenic effects. Acrylamide has been implicated in genetic mutation formation and tumorigenesis in various organs.^24^ In this study, we aimed to investigate the effects of exposure to ACR on kidney tissue using histopathological and stereological methods. Another aim was to assess the immunoexpression of PlGF in a model of probable toxicity and to investigate the therapeutic potential of OLE.

The principal finding was the detection of elevated PlGF immunoreactivity in the ACR exposure group and its amelioration by OLE. The stereological results also revealed an increased volume in distal and proximal tubules in the ACR group. Oleuropein treatment in ACR exposure (ACR + OLE group) also exhibited beneficial effects in terms of this volume increase.

Elevated PlGF levels have been detected in patients with reduced kidney function.^25^ Moreover, many other cell types such as keratinocytes, cardiomyocytes, retinal pigment epithelial cells, bronchial epithelial cells, and tumor cells have been reported to express PlGF in pathological conditions.^26^ Placental growth factor stimulates pathological angiogenesis by upregulating tumor necrosis factor α and monocyte chemotactic protein-1.^27^ The findings of the present study also confirm that PlGF-immunopositive cells are highly expressed in ACR-exposed kidney tissue. The main conclusion of this study is that PlGF overexpression accompanies ACR nephrotoxicity.

Stereological analyses allow reliable numerical data to be obtained from tissue sections. Pathological changes in structures can also alter their numerical properties, and quantitative analyses, therefore, permit predictions to be made about structures. The volume increase in the cortex in the ACR group in the present study may be associated with the volume increase in the distal and proximal tubules. Our histological results also support the stereological results, since we detected enlargement in the proximal and distal tubules. Consistent with the present research, other previous studies have confirmed an increase in renal tubule volume due to various toxicity patterns.^28^ Karimfar et al^29^ showed dilation in distal and proximal tubules in rabbits following prolonged exposure to lead acetate. Acrylamide toxicity causes mitochondrial swelling in renal tubular cells and impairs energy production, which may be another reason for increased tubular volume. Additionally, in the present study, no significant difference was found between the groups in terms of glomerular volume. Changes in glomerular volume have been reported to generally begin with long-term exposure to toxic agents. The absence of change in glomerular volume in our study, in contradiction to the changes observed in other kidney structures, may be due to the different physiological and histological conditions of the glomeruli compared to other renal tissues, which increases their resistance to toxins compared to other tissues. Glomeruli are also known to possess a defensive barrier due to their unique structure.

Placental growth factor overexpression may also explain the volume increase in kidney structures, because the volume findings in the ACR + OLE group, in which PlGF expression was partially reduced, were similar to those in the control group. A previous study detected decreased cortex and medulla volumes and glomeruli numbers in neonatal rat kidneys in an ACR-exposed group.^30^ These results that are inconsistent with those of the present study may be due to the analyses being conducted on neonatal rat kidneys. Acrylamide exposure may be acting through different mechanisms during fetal development.

A similar intensity of PlGF immunoreactivity in glomeruli was observed between the groups. Following this, no difference was found in terms of glomerular volume between the groups, supporting our view that there may be a connection between PlGF and volumetric changes.

Histopathological studies have reported that ACR exposure caused shrinkage of the glomeruli, loss of proximal tubule brush borders, degeneration of the kidney epithelium, accumulation of necrotic areas in the renal parenchyma, and increases in p53 expression and caspase-3 activity in kidney tissue.^9^ Another study reported glomerular collapse, narrowing of Bowman’s spaces, and damage and tubular degeneration in proximal and distal tubular epithelial cells in kidney sections from a group treated with ACR.^7^ These studies have proved that exposure to ACR causes toxic effects by adversely affecting enzymatic antioxidant status (superoxide dismutase (SOD), catalase (CAT) and glutathione peroxidase (GPx) activities) and increasing oxidative stress in kidney tissue.^31^

The present study showed various pathological conditions, such as tubular dilatation, vacuolization and degeneration in proximal and distal tubules, necrotic distal tubule epithelial cells, and occlusions in capillaries. Free radicals can lead to the degradation of polyunsaturated fatty acids in plasma membranes, resulting in vacuolization. In addition, vacuolization, which can often occur with exposure to toxins, may perform the task of collecting and isolating harmful elements as a kind of protective defense mechanism against harmful effects. Inflammatory responses from reactive oxygen species (ROS) can cause vascular congestion.^8^ All these findings confirm that ACR is highly toxic to kidney tissues. This study investigated the effects of OLE against these damages and indicated that this exhibits renoprotective potential in ACR exposure.

Oleuropein is both an anti-proliferative and an apoptotic molecule. The protective effects of OLE on kidney damage studied by various experimental models.^32,33^ A recent study revealed that OLE is capable of attenuating the excessive production of ROS induced by cigarette smoke in kidney and liver tissues.^32^ In another study, OLE exhibit beneficial effects on cisplatin-induced hepatotoxicity, nephrotoxicity, immunotoxicity, and genotoxicity by ameliorating oxidative stress, inflammation, and apoptosis.^34^

Mohammed et al^35^ investigated the effects of olive leaf extract on the kidneys of offspring exposed to maternal diabetes and reported a curative effect on kidney functions due to its antioxidant property. Another study reported that OLE (100 and 200 mg/kg) exerted a protective effect against kidney damage caused by unilateral ureteral obstruction through antioxidative, anti-apoptotic, and anti-inflammatory mechanisms.^36^ Hematoxylin and eosin staining findings in that study showed that OLE reduced glomerular or tubulointerstitial damage.^36^ Similarly, the histopathological findings of the present study found that OLE exerts a therapeutic effect against vacuolization and degeneration in tubules, and our stereological results in terms of volume analysis proved that OLE treatment normalized the proximal and distal tubules. We also found that, for the first time in the literature, OLE reduced PlGF-immunopositive cell intensity, a finding which may be attributed to its antioxidant properties, although we did not measure the oxidative parameters. In this case, biochemical studies are needed to evaluate the antioxidant effects of OLE on the kidney. Although these studies exist in the literature, the fact that we did not do biochemical analysis is a limitation of our study. It is also another shortcoming that the mRNA expression levels of PlGF have not been investigated.

## Conclusion

In conclusion, our findings support the view that exposure to ACR results in alterations to renal histopathology. Increased PlGF levels were found to be correlated with kidney injury.

Oleuropein attenuated elevated PlGF immunoreactivity and volume increases in the cortex and proximal and distal tubules in ACR-exposed kidney tissue. The findings of this study revealed an association between PlGF and volumetric changes in kidney tissue. Placental growth factor may, therefore, be recommended as an indicator of renal toxicity in ACR exposure. The molecular mechanism by which PlGF achieves this now needs to be investigated.

## Figures and Tables

**Table 1.** Anti-PlGF Staining Intensity in All 4 Groups

Parameters	Groups			
	Control	OLE	ACR	ACR + OLE
Distal tubules	+	+	+	+
Proximal tubules	++	++	+++	++
Glomeruli	+	+	+	+
Medulla	+	+	+	+

ACR, acrylamide; OLE, oleuropein.

**Table 2.** Anti-PlGF Staining H-scores in All 4 Groups

	Groups			
	Control	OLE	ACR	ACR + OLE
H- score	163 ± 8.2^b,c,d^	181 ± 15^a,c^	352 ± 9.4^a,b,d^	198 ± 8.7^a,c^

ACR, acrylamide; OLE, oleuropein.

^a,b,c,d^Different from the control, OLE, ACR, and ACR + OLE groups, respectively (*P* < .01).

**Table 3:** Statistical Expression of the Total Distal and Proximal Tubule, Glomerulus, Cortex, and Medulla Volumes Between the Control and Experimental Groups

Volume (Mean ± SD), mm^3^				
Parameters	Groups			
	Control	OLE	ACR	ACR + OLE
Distal tubules	165 ± 3.7^b,c^	215 ± 22^a,d^	240 ± 3.1^a,d^	163 ± 4.9^b,c^
Proximal tubules	983 ± 117^c^	938 ± 66^c^	1583 ± 46^a,b,d^	1015 ± 89^c^
Glomeruli	63 ± 7.7	64 ± 7.4	63 ± 17.2	73 ± 14
Cortex	1018 ± 44^b,c,d^	1478 ± 63^a,c,d^	1982 ± 239^a,b,d^	1625 ± 53^a,b,c^
Medulla	520 ± 9.7^b,c^	260 ± 7.2^a^	191 ± 68^a,d^	409 ± 108^c^

ACR, acrylamide; OLE, oleuropein.

^a,b,c,d^ Different from the Control, OLE, ACR, and ACR + OLE groups, respectively (*P* < .01).
